# Induction of Systemic Resistance in Maize and Antibiofilm Activity of Surfactin From *Bacillus velezensis* MS20

**DOI:** 10.3389/fmicb.2022.879739

**Published:** 2022-05-09

**Authors:** Shireen Adeeb Mujtaba Ali, R. Z. Sayyed, Mohammad I. Mir, M. Y. Khan, Bee Hameeda, Mustfa F. Alkhanani, Shafiul Haque, Abdel Rahman Mohammad Al Tawaha, Péter Poczai

**Affiliations:** ^1^Department of Microbiology, University College of Science, Osmania University, Hyderabad, India; ^2^Department of Microbiology, PSGVP Mandal’s Arts, Science, and Commerce College, Shahada, India; ^3^Department of Botany, University College of Science, Osmania University, Hyderabad, India; ^4^Kalam Biotech Pvt Ltd., Hyderabad, India; ^5^Emergency Service Department, College of Applied Sciences, Al-Maarefa University, Riyadh, Saudi Arabia; ^6^Research and Scientific Studies Unit, College of Nursing and Allied Health Sciences, Jazan University, Jazan, Saudi Arabia; ^7^Bursa Uludağ University Faculty of Medicine, Bursa, Turkey; ^8^Department of Biological Sciences, Al-Hussein Bin Talal University, Ma’an, Jordan; ^9^Finnish Museum of Natural History, University of Helsinki, Helsinki, Finland

**Keywords:** *Bacillus velezensis* MS20, biocontrol, biosurfactants, characterization, induced systemic resistance, optimization-OVAT, sustainable agriculture, antibiofilm

## Abstract

Surfactin lipopeptide is an eco-friendly microbially synthesized bioproduct that holds considerable potential in therapeutics (antibiofilm) as well as in agriculture (antifungal). In the present study, production of surfactin by a marine strain *Bacillus velezensis* MS20 was carried out, followed by physico-chemical characterization, anti-biofilm activity, plant growth promotion, and quantitative Reverse Transcriptase—Polymerase Chain Reaction (q RT-PCR) studies. From the results, it was inferred that MS20 was found to produce biosurfactant (3,300 mg L^–1^) under optimized conditions. From the physicochemical characterization [Thin layer chromatography (TLC), Fourier Transform Infrared (FTIR) Spectroscopy, Liquid Chromatography/Mass Spectroscopy (LC/MS), and Polymerase Chain Reaction (PCR) amplification] it was revealed to be surfactin. From bio-assay and scanning electron microscope (SEM) images, it was observed that surfactin (MIC 50 μg Ml^–1^) has appreciable bacterial aggregation against clinical pathogens *Pseudomonas aeruginosa* MTCC424, *Escherichia coli* MTCC43, *Klebsiella pneumoniae* MTCC9751, and Methicillin resistant *Staphylococcus aureus* (MRSA) and mycelial condensation property against a fungal phytopathogen *Rhizoctonia solani*. In addition, the q-RTPCR studies revealed 8-fold upregulation (9.34 ± 0.11-fold) of *srf*A-A gene compared to controls. Further, treatment of maize crop (infected with *R. solani*) with surfactin and MS20 led to the production of defense enzymes. In conclusion, concentration and synergy of a carbon source with inorganic/mineral salts can ameliorate surfactin yield and, application wise, it has antibiofilm and antifungal activities. In addition, it induced systemic resistance in maize crop, which makes it a good candidate to be employed in sustainable agricultural practices.

## Introduction

Lipopeptides biosurfactant are non-ribosomal peptides that are produced extracellularly during the stationary phase in the presence of various carbon and nitrogen sources (Armas et al., 2019). Numerous superlative activities of lipopeptides have sparked researchers’ considerable interest to explore effective ways for increased yield. Several studies on the impact of environmental factors on lipopeptide biosynthesis have shown that composition and yield of lipopeptide mixture may be influenced by media, nutrition sources, and growth conditions (temperature, pH, and oxygen) (Hmidet et al., 2017). Parallelly, papers on biosurfactant production in the presence of various nutritional sources and limiting environmental conditions are available. For example, Agarwal and Sharma (Agarwal and Sharma, 2009) demonstrated the effects of various Carbon sources on biosurfactant synthesis, including glycerol, molasses, rice water, cheese whey, potato peels, and glucose.

Biosurfactants are widely used as antagonistic molecules against pests/pathogens or plant diseases and have been used to improve soil quality by decomposing toxic and hazardous pollutants or making trace nutrients available in the soil for sustainable agricultural methods. The antibacterial properties of surfactants generated by microbial strains significantly suppress pathogen growth. It defends the plant from pathogen infection in certain circumstances by boosting the immune system of the plant, stimulates rhizosphere microflora, and maintains the physiological parameters of plant (Vatsa et al., 2010). When compared to conventional antimicrobial agents or pesticides, they can infiltrate and damage fungal cell membranes and lower the probability of resistance (Choub et al., 2021). Cyclic lipopeptides (from *B. velezensis*) are demonstrated to inhibit fungal growth (Akladious et al., 2019). They are potential biocontrol agents against a variety of fungal plant diseases. Among these, surfactin lipopeptide biosurfactant is useful as a biopesticide component because of its temperature and pH stability, as well as its biodegradability and low toxicity. It is reported for its ISR (induced systemic resistance) properties and use in sustainable agriculture (Théatre et al., 2021). The mechanism of the surfactin is explained as it enters cell bilayers as an antibacterial agent, chelates cations, and solubilizes membranes and lyses pathogens by pore creation (Li et al., 2021).

Repeated studies have shown that biosurfactants have the capacity to prevent and disrupt biofilms, such as rhamnolipids’ ability to decrease viable bacteria (3–4 log reduction) (Staudt et al., 2004). For example, around 90% biofilm inhibition and 65% disruption in *Streptococcus sanguinis* has been reported; similar studies on disruption and antimicrobial property of sophorolipid (5%) against *Bacillus subtilis* BBK006 and *Cupriavidus necator* ATCC 17699 are also demonstrated (Díaz De Rienzo et al., 2015). Surfactin from *Bacillus circulans* is an example of a propitious lipopeptide with antimicrobial property (Das et al., 2008). A recently published research article demonstrated antibiofilm property of two biosurfactants (rhamnolipids and surfactin) (Yamasaki et al., 2020).

Maize is a major cereal crop that is cultivated for food, feed, and fuel all over the world. Biological and abiotic stressors commonly impact its production, causes reduced yield and quality, and interferes with the maximum yield potential. Banded leaf and sheath blight (BLSB), caused by *Rhizoctonia solani*, is a new and severe infection that restricts crop output in climatic situations, especially with monsoons in India. *R. solani* colonizes aerial plant parts and produces phytotoxins, which are responsible for the formation of necrotic spots on stem, leaf, and sheath (Singh et al., 2020).

Surfactin can help with biocontrol even if pathogens are not lysed because of its role in *Bacillus* biofilm formation. This biofilm development can disrupt cohabitant pathogen biofilm and also cause systemic resistance in plants. In addition, surfactin is reported to stimulate production of defense enzymes (phenylalanine ammonia lyase) in tobacco plant cells and have no phytotoxicity (Jourdan et al., 2009).

In the present study, optimization of surfactin production was carried out by one variable at a time (OVAT) approach, followed by characterization of compound by TLC, FTIR, and LC/MS, assayed for biofilm inhibition against clinical pathogens (*P. aeruginosa* MTCC424, *E. coli* MTCC43, *K. pneumoniae* MTCC9751, and MRSA) and anti-fungal activity against *Rhizoctonia solani*. Quantitative real-time polymerase chain reaction (q RT-PCR) was done in order to study % up-regulation or down-regulation of *srfA-A* genes in the presence and absence of MgSO_4_ and glucose. Furthermore, biocontrol efficacy of MS20 and surfactin was also assessed.

## Materials and Methods

### Production, Extraction, Characterization, and Purification of Biosurfactant

For the production of biosurfactant, 2% of actively grown overnight culture of *B. velezensis* MS20 GenBank accession number LR535811 (Ramavath et al., 2019) was inoculated in 100 mL nutrient broth amended with 0.5 % (w/v) different inorganic/mineral salts like MgSO_4_, KNO_3_, Fecl_3_, and Mncl_2_ and 2 % (w/v) different carbon sources i.e., Glucose, Maltose, Fructose, Sucrose (in different combinations) in 250 mL centrifugation at 15,000 rpm for 15 min at 4°C. Cell free supernatant was subjected to acid precipitation with 6N HCl and dried by rota evaporation as described previously (Long et al., 2017; Liu et al., 2020).

The above concentrated biosurfactant was dried, weighed, reconstituted in methanol, filtered through 0.22 μ pore size syringe filters, and used for TLC analysis as described by Parameshwar et al. (2019) and Li et al. (2021) with slight modification. Silica gel 60 F254 (Merck Co., Darmstadt, Germany)-coated aluminum plates were used with the help of capillary tube, wherein a drop of crude extract was placed on silica gel plates, dried, and kept in chromatography chamber with mobile phase i.e., methanol: chloroform: water (65:25:4) v/v. The presence of biosurfactant was detected by 0.1% ninhydrin in acetone. Retardation factor (R_f_) value was calculated by formula –


$$\text{Rf}=\frac{D\,i\,s\,t\,a\,n\,c\,e\,t\,r\,a\,v\,e\,l\,l\,e\,d\,b\,y\,s\,o\,l\,u\,t\,e}{D\,i\,s\,t\,a\,n\,c\,e\,t\,r\,a\,v\,e\,l\,l\,e\,d\,b\,y\,s\,o\,l\,v\,e\,n\,t}$$

Solute: stationary phase: lipopeptide sample

Solvent: mobile phase

FTIR analysis of the above crude extract was done by FTIR spectrophotometer (Shimadzu Co., Japan) with rota evaporated and dried 1 mg of crude extract in pellets of potassium bromide. IR spectra was obtained in a range of 1,000–3,500 cm^–1^ with a spectral resolution of 4 cm^–1^ (Parameshwar et al., 2019).

Around 10 mg of crude extract (from above) was reconstituted in methanol and filtered by 0.22 μ pore size syringe filters. Of this filtered biosurfactant, 20 μl was injected in RP-HPLC (SPD-20A, Shimadzu Co., Japan) and collected several times by reinjection of filtered biosurfactant. Purified biosurfactant was further used for characterization by ESI/MS and bioassays. The protocol followed for RP-HPLC was as described by Parameshwar et al. (2019). In brief, 20 μl filtered sample was injected into RP- HPLC (shimadzu SPD-20A Japan) with column:C18 (4.6 mm × 250 mm, 5 μm, Agilent, Santa Clara, CA, United States), for mobile phase solvent A- 0.1% (triflouroacetic acid) of 90% methanol at a flow rate of 1 mL min^–1^ at an elution time 0–30 min; UV absorbance at 210 nm was maintained for this study. 20 μl of purified biosurfactant was further subjected for MS analysis by mass spectrometry connected with an ESI source. Spectra were recorded at positive and negative polarities.

For the detection of *srf*AB gene by PCR amplification, genomic DNA from *B. velezensis* MS20 was isolated by conventional phenol-chloroform method (Koons et al., 1994). *srfAB* gene specific primers with sequence Forward primer: 5-TTTACTCATACTA CGTCAAC-3′, Reverse Primer: 5-GTGTATTAAGAAATTCG AGC-3′ (Swapna et al., 2016) were used in this study. PCR amplification was carried out in a 20 μl reaction mixture comprising DNA template (2 μl), 10 μl of PCR master mix, and 4 μl nuclease free water. Amplification was done in Eppendorf AG, Mastercycler Nexus Series. The PCR amplification protocol for this work is as follows: initial denaturation at 94°C for 2 min, 30 cycles of 95°C for 3 min denaturation, annealing 46°C for 2 min, extension 72°C for 2 min, elongation 72°C for 5 min. PCR amplified product was subjected to gel electrophoresis with 1% agarose gel and results were observed on Gel DOC system (Bio-RAD, Gel DOC, EZ IMAGER, United States).

### Antibiofilm Assays

Aggregation assay was performed as described by Xiu et al. (2018) with some modification. In brief, 100 μl of 1:100 dilution of overnight grown clinical pathogens [(*P. aeruginosa*, *E. coli*, *K. pneumoniae*) and MRSA (obtained from a local hospital)] in Luria Burtani (LB) broth with 100 μl surfactin (1:1) at concentration 50 and 100 μg mL^–1^ was added in 96 wells polystyrene titer plate aseptically, as well as 200 μl of active culture which was considered as control. After incubation for 24 h, wells were washed twice with sterile distilled water, air dried, and fixed with 100 μl methanol for 15–20 min. Again, wells were rinsed with sterile distilled water and crystal violet assay was performed. To this, 200 μl of 0.1% crystal violet (CV) was added, kept static for 20 min, then washed with distilled water, air dried for 30 min at 28°C, and photographed.

Anti-adhesion assay was performed as described by Rodrigues and Campos-Takaki (2011) with some modification. In brief, 96 wells polystyrene titer sterile plates were inoculated with 200 μl of purified extract (50 and 100 μg mL^–1^ concentration) and incubated for 22–24 h at 4°C. Then, wells were washed twice with phosphate buffer (PB) (pH 7), air dried at room temperature, 200 μl of diluted (as mentioned above) pathogenic bacterial cultures were added, and 200 μl of active culture was considered as control and incubated for 4 h at 37°C. Again, plates were washed with PB. Then bacterial cells were fixed with 200 μl of methanol for 15 min, and wells were emptied and dried followed by quantification by CV assay. Wells were stained with 200 μl of 2% CV for 5 min, then washed in tap water, air dried, and resolubilized with 200 μl of 33% glacial acetic acid.

Samples for SEM analysis were prepared as described by Xiu et al. (2018) with some modification. In brief, an overnight grown MRSA culture was diluted 1:100 times in Luria Bertani broth and incubated for a further 3–4 h at 37°C and 150 rpm until it attained a cell density of 0.2–0.3 OD_600_. Four samples were prepared from cell suspension by addition of sterile distilled water, methanol, and surfactin (50 and 100 μg mL^–1^ concentration, respectively) and incubated for 3–4 h. Simultaneously, grease-free cover slips were overlaid with 1% gelatin, and cell suspension (treated with surfactin) was added as a drop over coverslips, and allowed to dry. Cells were then fixed with 5% glutaraldehyde for 1 h. Then cover slips were dehydrated with an ethanol gradient of 50, 60, 70, and 80% with 10 min of incubation for each gradient and analyzed by SEM, from which images were generated.

### Antagonistic Studies

A loopful of overnight grown culture of *B. velezensis* MS20 on Luria Bertani broth (LB) was streaked on a potato dextrose agar (PDA) plate pre-inoculated at the center of the plate with 6 mm diameter *Rhizoctonia solani* fungal plug and incubated at 25°C for 48 h. Fungal mycelium faced toward the bacterial colony was picked with sterile forceps and teased on a microscopic glass slide with a drop of lactophenol cotton blue. A clean cover slip was kept on this and observed under light microscope at 40× objective. Pictures were taken with a Nikon P310 digital camera. Fungal mycelium from the same plate were used for SEM analysis. Antifungal activity of MS20 was also assessed in PD broth (PDB). A 250 mL Erlenmeyer flask with 100 mL PDB was inoculated with 6 mm diameter *R. solani* fungal plug and incubated for 24 h at 25°C. Then 2% MS20 culture was added and incubated for a further 48 h. PDB with only fungal culture was considered as control. After 72 h of incubation, broth was filtered through Whatmann filter paper 1, fungal biomass was collected, dried in incubator overnight, and the weight was recorded.

Antifungal activity of purified extract was also determined by agar well assay on 24 h pre-inoculated PDA plate with *R. solani*. Plates were incubated at 25°C temperature for 48 h. Growth was calculated as average of triplicates. Mycelial growth inhibition (MGI) was calculated by formula (Teixeira et al., 2021).


MGI=C-TC⁢x⁢ 100

Where MGI = Mycelial growth inhibition, C = control, T = Test.

### Analysis of *srf*A-A Gene Expression by q-RTPCR

*B. velezensis* MS20 was grown in nutrient broth (NB) at 37°C for 48 h supplemented with (1) NB medium with only 0.5% MgSO_4_, (2) NB medium with only 2% Glucose, or (3) NB medium with 2% glucose and 0.5% MgSO_4_. NB medium inoculated cells without MgSO_4_ and glucose were used as controls. Total RNA from *B. velezensis* MS20 was extracted by NucleoSpin RNA kit (Macherey-Nagel, Duren, Germany) in compliance with the manufacturer’s directions. Quantity and quality of RNA samples were assessed by NanoDrop (Thermo Fisher Scientific) and 1.5% (w/v) agarose gel. Further, 5 μg of total RNA was used for complimentary DNA (cDNA) synthesis by PrimeScript 1st strand _C_DNA synthesis kit (cat. 6110A Takara). Expression levels of genes involved in surfactin lipopeptide synthesis in *B. velezensis* MS20 were characterized by Quantitative-PCR (qRT-PCR) by a Mastercycler (Step one Plus Real Time PCR Applied Biosystem Invitrogen Bioservices India Pvt. Ltd, CA, United States). Table 1 displays primers which were used for amplification of specific genes in surfactin lipopeptide synthesis and 16S rDNA gene was used as an internal control. SYBR, Premix Ex Taq™ II (Cat. RR820A Takara) were used for PCR cycle. RT-PCR mixture (20 μl) taken was as follows: 10 μl of 50X SYBR Premix Ex Taq (Takara), 2 μl of cDNA template, 1.6 μl of mixed PCR forward and reverse primers (10 μm), and 6.4 μl of DEPC treated water. For both control group and evaluation group, three separate samples were measured. Amplification of target DNA was attained with initial cDNA denaturation at 95°C for 00:30 min, 40 cycles that comprised denaturation for 00:05 s at 95°C, 00:40 s at 51°C for primer annealing, and 1:00 min at 60°C for primer extension. 2^–ΔΔCT^ (minus of delta) delta curve threshold approach was used for analysis of relative changes from real-time PCR experiments in surfactin lipopeptide gene expression (Ding et al., 2018).

**TABLE 1:** *SrfA-A* gene primers and 16S rRNA primer sequence.

Primers	Sequence	References
*Srf A-A*-F	5′-GCCTATGTGCCGATTGAT-3′	Ding et al. (2018)
*SrfA-A*-R	5′-ATGCTGGATTGTGAGAGTC-3′	Ding et al. (2018)
16S r RNA-F	5′-CCACACAGGGACTGAGACAC-3′	Ding et al. (2018)
16S r RNA-R	5′-ACTTAAGAAACCGCCTGCGA-3′	Ding et al. (2018)

### Plant Biocontrol Experiment

Maize seeds (local variety) were purchased from open market Madannapet Mandi Hyderabad Telangana India. Seeds were surface sterilized with 1% sodium hypochlorite (NaOCl) for 1 min followed by washing several times with sterile distilled water. Surface sterilized maize seeds were coated with the following treatments T1: *Bacillus velezensis* MS20 in 1% carboxy methyl cellulose (CMC); T2: Surfactin; T3: Fungicide; T4: *Bacillus subtilis* MTCC 2424; T5: Uninoculated NB; and T6: Treated with only fungi. 10 pre-treated seeds were then sown in pots with 5 kg soil.

The experimental design comprised six different treatments in triplicates and the pots were maintained in green house conditions for a period of 30 days at a temperature of 26°C and humidity of 80–90%. As soon as seed germination started, pathogen inoculation was done, i.e., *R. solani* inoculum prepared in rice husk was added in close contact with roots.

After 15 days, pathogen inoculation (DAPI) maize leaves and roots from each treatment were sampled to assess total chlorophyll, total carotenoids content, total sugar, protein, proline, and H_2_O_2_ at 0, 6th, and 12th DAPI as per the methodology of Sadasivam and Manickam (1996) and Thimmaiah (2012). Followed by quantitative estimation for phenylalanine ammonia lyase (PAL), ascorbate peroxidase (APx), peroxidase (POx), and catalase (CAT) was performed (Singh et al., 2020) at 0, 6th, and 12th DAPI. For estimation of the activity of phenylalanine ammonia lyase (PAL), tissue sample (1 g) was grounded in 4 mL 0.2 M borate buffer (pH 8.7) with 1.4 mM β-mercaptoethanol; this enzyme extract (200 μl) was used for assay wherein L-phenylalanine and cinnamic acid were used as substrates, and PAL was determined spectrophotometrically at 290 nm. Likewise, for peroxidase activity, 200 μl enzyme extract was used with guaiacol (20 mM) and H_2_O_2_ (12.3 mM), and absorbance was measured at 436 nm every 30 s for 3 min. Estimation of ascorbate peroxidase was performed with enzyme extract and ascorbic acid (10 mM) added as substrate; absorbance was measure at 265 nm every 30 s for 5 min. Catalase activity was determined with H_2_O_2_ (2.5 mM) and enzyme extract. Activity was assessed by spectrophotometer at 240 nm for 1 min through degradation of H_2_O_2_. Chitinase and superoxide dismutase (SOD) activity were analyzed in plant leaves and roots as described by Thimmaiah (2012).

### Statistical Analysis

All experiments were performed in triplicate and mean was calculated. Normality was checked by Shapiro Wilks Test. Student’s *t*-test was performed to check the probability and one way ANOVA after log transformation and 95% confidence intervals was used for statistical analysis with significance level of *P* < 0.05 in comparison with controls.

## Results

### Production, Extraction, Characterization, and Purification of Biosurfactant

**FIGURE 1:**
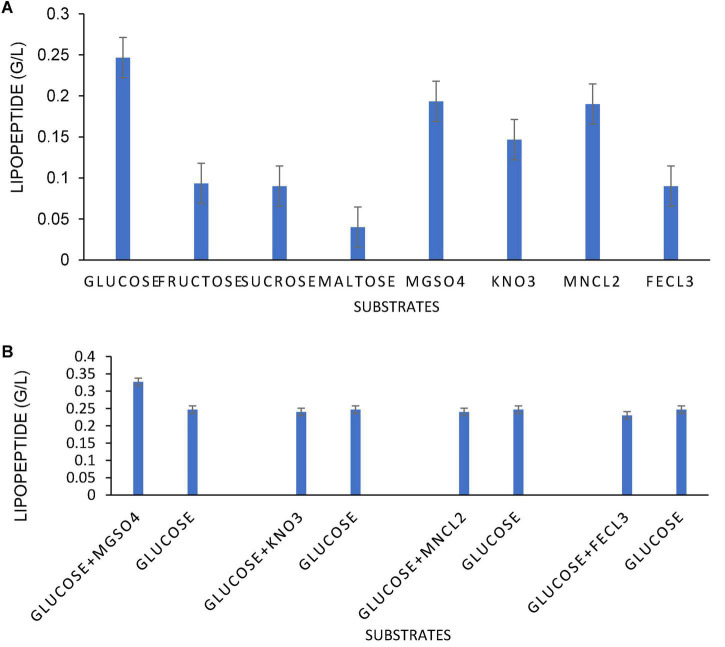
Optimization of different (0.5% w/v) inorganic salts and (2% w/v) Carbon sources for lipopeptide (surfactin) production **(A)** Lipopeptide production in g/L in presence of 2% w/v C sources and 0.5% w/v inorganic salts (Individually). **(B)** Lipopeptide production in g/L in presence of 2% w/v C sources and 0.5% w/v inorganic salts (In synergy).

**FIGURE 2:**
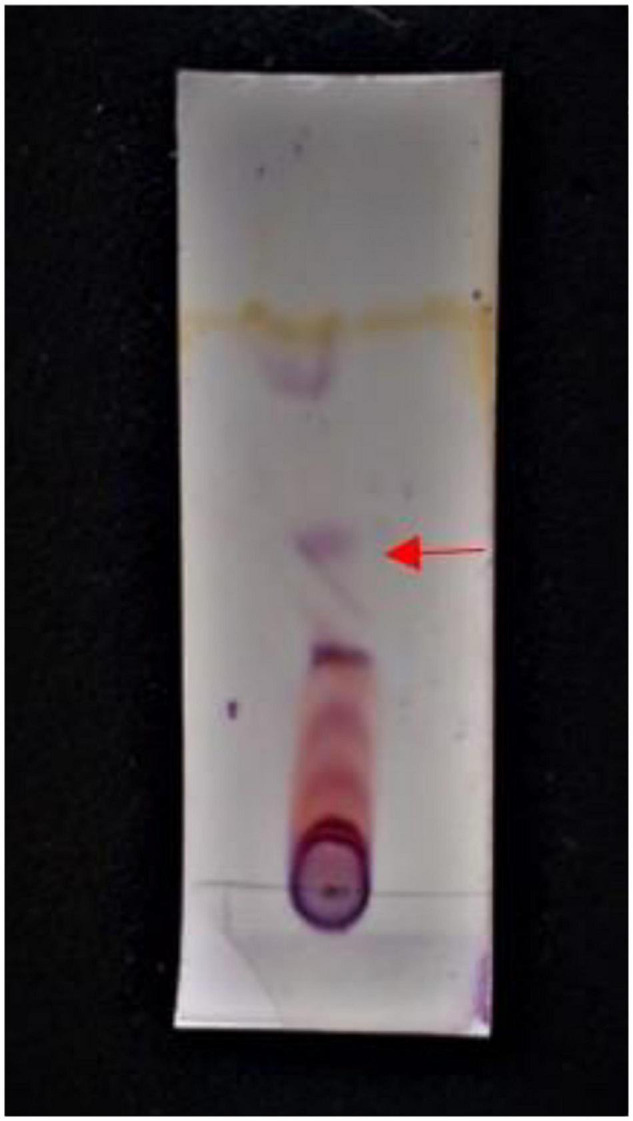
TLC plate showing band at Rf value 0.7 (Swapna et al., 2016; Parameshwar et al., 2019; Ramavath et al., 2019).

**FIGURE 3:**
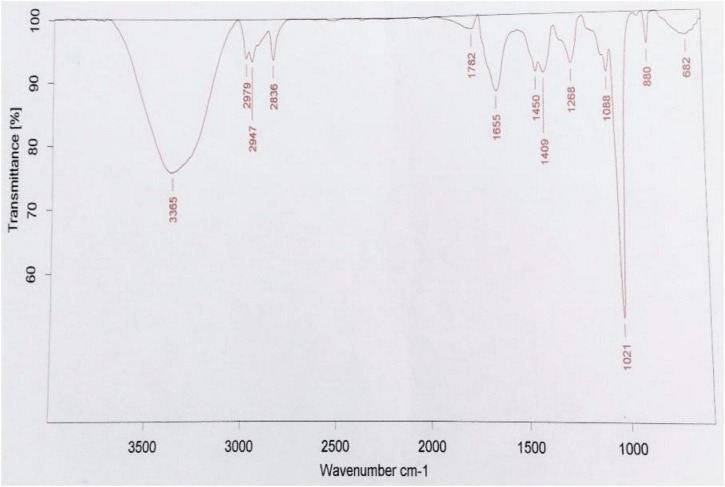
FTIR spectra of Surfactin Lipopeptide of *B. velezensis* MS20 (Parameshwar et al., 2019).

The optimization of media was carried out in a series of experiments changing one variable at a time, keeping the other factors fixed at a specific set of conditions. The results of media optimization for biosurfactant production revealed the highest production of biosurfactant i.e., 3,300 mg L^–1^, when NB was inoculated with 2% of MS20, amended with 0.5% MgSO_4_ and 2% Glucose after 48 h of incubation period (Figure 1). Primary characterization of extracted biosurfactant from MS20 was analyzed by TLC silica gel plate. Upon exposure to 1% ninhydrin, the appearance of a pink spot was noticed with R_f value_ 0.7 (Figure 2) and PCR amplification of *srf*AB gene resulted in 675 bp fragment (Supplementary Information 1) which confirmed the presence of surfactin. Furthermore, the presence of functional groups in the biosurfactant produced by MS20 was determined by FTIR that revealed C and N-H stretches at 3,365 cm^–1^. Also revealed was an aliphatic chain and C-CH_3_ bond at 2,836–2,979 cm^–1^. Absorbance at 1,782 cm^–1^ showed the presence of carbonyl group or lactone ring. A peak at 1,655–1,782 cm^–1^ depicted the presence of peptide and deformed N-H and C-N stretches at 1,450 cm^–1^ (Figure 3). Purified extract was analyzed by ESI-MS at positive and negative polarity which showed four characteristic peaks corresponding to isoforms that are in accordance with literature reported for surfactin (Table 2).

**TABLE 2:** Detection of surfactin lipopeptide by LC/ESI/MS.

Product Ion Negative	Product Ion Positive	Negative m/z	Positive m/z	Molecular weight/Exact mass	Compound	References
[M-H]^–^	[M+H]^+^	982.3	960.4	93	C12	Sarwar et al., 2018; [Bibr B27][Bibr B13]
		839.9	1007.1	1007	C13	
		1012.8	927.9	1021	C14	
		1014.1	1033.7	1035	C15	
		1059.3	1079.7	1049	C16	
		–	–	1063	C17	

### Antibiofilm Assays

**FIGURE 4:**
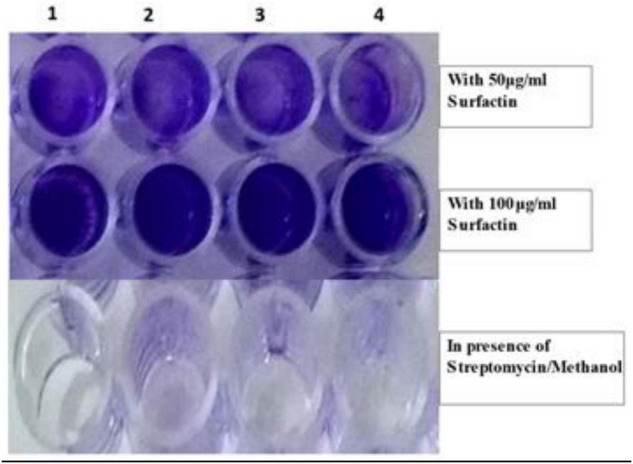
Aggregation of clinical pathogens in the presence of Surfactin (at 50 and 100 μg ml^–1^ concentration) and in its absence. (1) *P. aeruginosa* MTCC424, (2) *E. coli*MTCC43, (3) *K. pneumoniae*MTCC9751, and (4) MRSA.

**FIGURE 5:**
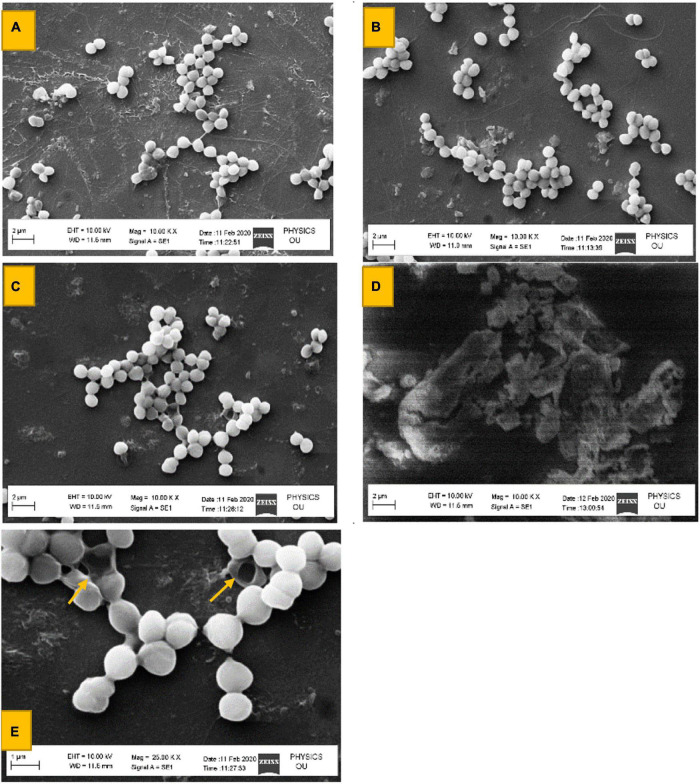
SEM Images of MRSA cell at 2 and 1 μm. **(A)** Only sterile distilled water 2 μm. **(B)** Cell suspension with 50 μl of methanol (2 μm). **(C)** Cell suspension treated with 50 μl of 50 μg ml^–1^ Surfactin (2 μm). **(D)** Cell suspension treated with 50 μl of 100 μg ml^–1^ Surfactin (2 μm). **(E)** Cell suspension treated with 50 μl of 50 μg ml^–1^ Surfactin at (1 μm).

From bioassays, aggregation of clinical pathogens *P. aeruginosa* MTCC424, *E. coli* MTCC43, *K. pneumoniae* MTCC9751, and MRSA in 96 well microtiter plates at MIC 50 μg mL^–1^ was observed (Figure 4) which suggests that surfactin can act as an anti-biofilm agent by restricting the motility of pathogens and preventing the formation of biofilm. In continuation, SEM images of MRSA revealed visible aggregation in comparison to its respective control i.e., no change in MRSA cells treated with sterile distilled water and methanol, and clear cell wall disruption and aggregation at 50 and 100 μg mL^–1^ concentration, respectively (Figure 5).

Therefore, our results demonstrate visual evidence of condensation of *R. solani* mycelium and aggregation of MRSA in the presence of MS20 and surfactin, at 50 and 100 μg mL^–1^ concentration.

### Antagonistic Activity

**FIGURE 6:**
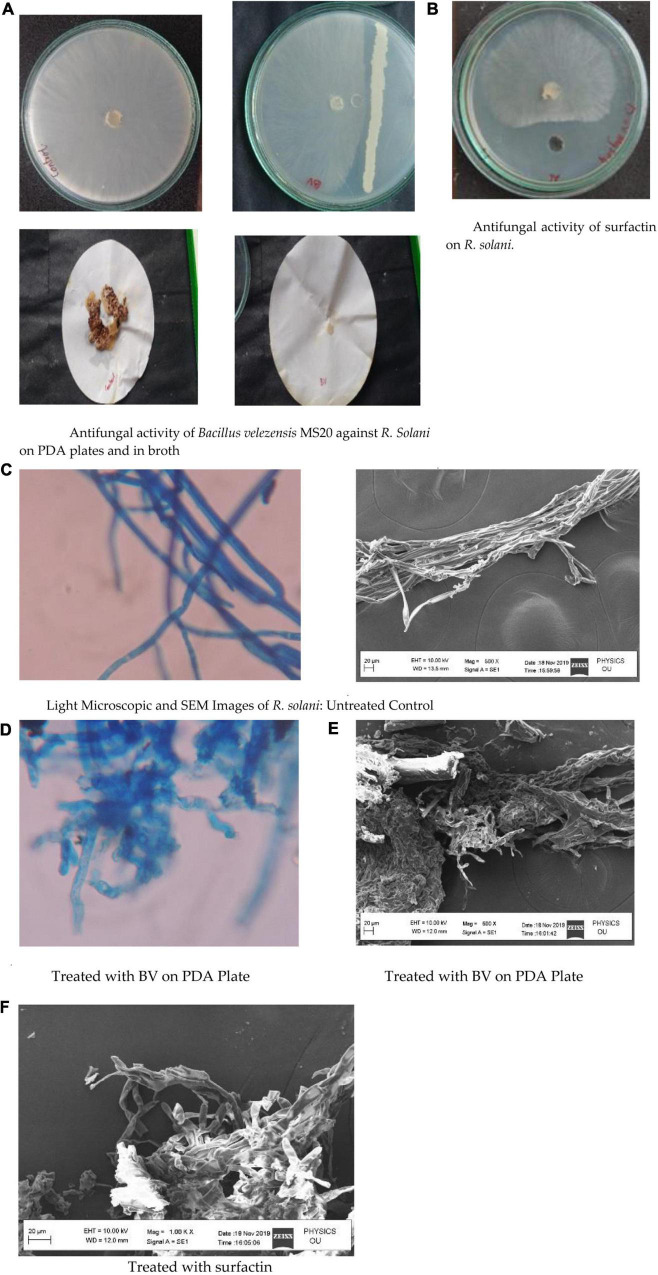
**(A)** Antifungal activity of *Bacillus velezensis* MS20 against *R. Solani* on PDA plates and in broth. **(B)** Antifungal activity of surfactin on *R. solani*. **(C)** Light Microscopic and SEM Images of *R. solani*: Untreated Control. **(D)** Treated with BV on PDA Plate. **(E)** Treated with BV on PDA Plate. **(F)** Treated with surfactin.

MS20 on PDA plate after incubation in comparison with control exhibited an inhibition zone of ∼40%, whereas in PD broth no fungal mycelium was detected. Simultaneously, surfactin exhibited antifungal activity on PDA plate with an inhibition zone ∼40% at 50 μg mL^–1^ concentration (Figure 6). Hence, from the results it is inferred that MS20 as well as surfactin has an anti-fungal property. Light microscopic and SEM images of *R. solani* showed clear mycelial condensation by surfactin compared to untreated controls.

### Quantitative Reverse Transcriptase—Polymerase Chain Reaction *srf*A-A Gene Expression Analysis

**FIGURE 7:**
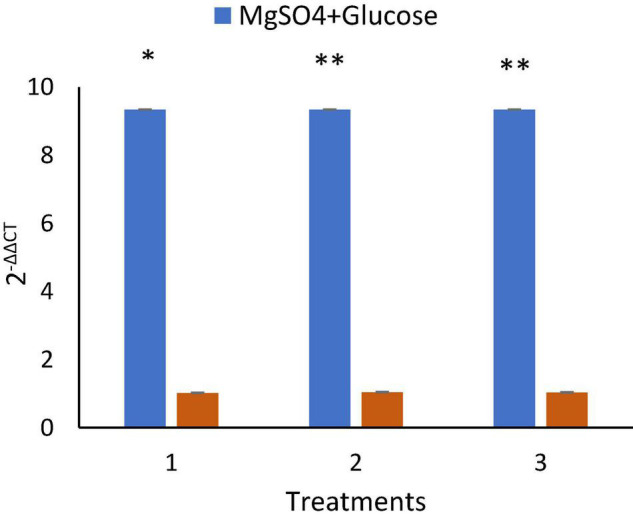
*B. velezensis* MS20 surfactin lipopeptide gene expression, showing 9.34 ± 0.11 upregulation in cells treated with MgSO4 and Glucose. By statistical analysis (*P* < 0.05), it was found to be highly significant. *P < 0.05, **P < 0.01.

Quantitative and qualitative analysis of RNA extracted was analyzed by Nanodrop (Table 3) and gel electrophoresis (Supplementary Information 2). Inoculation of MS20 in NB amended with 0.5% MgSO_4_ and 2% glucose resulted in upregulation of *srf*A-A gene to 9.34 ± 0.1-fold in q RT-PCR, whereas in untreated/control the expression levels were found to be 1.01 ± 0.1-fold, and 1.06 ± 0.1 in media amended with only 0.5% MgSO_4_ and 1.03 media amended with only 2% Glucose (Figure 7). Hence, our results demonstrate the surfactin gene upregulation under optimized conditions.

**TABLE 3:** Quantification of RNA by Nanodrop reading of RNA samples.

Sample Name	Concentration (ng/μ l)	260/280	260/230
Untreated	360.9	2.04	1.82
+MgSO_4_	500.7	2.10	2.26
+Glucose	325.6	2.16	2.02
+MgSO_4_+Glucose	503.2	2.14	1.99

### Plant Biocontrol Experiment

**FIGURE 8:**
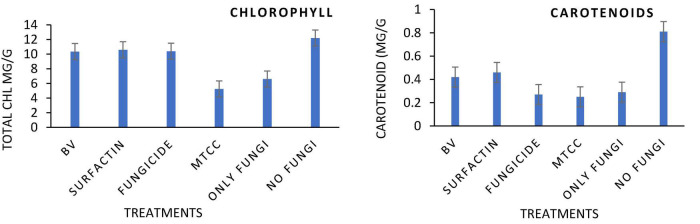
Effects of different treatments on Maize crop (a) total Chlorophyll content (b) total carotenoid content, at 15 Days After Pathogen inoculation DAPI and (g) H2O2 content in maize roots and shoot at 0, 6th, 12th DAPI under net house condition. Treatments: T1—BV: *B. velezensis* MS20 + *R. solani*; T2—Surfactin + *R. solani*; T3—Fungicide + *R. solani*; T4—*B. subtilis* MTCC2424 + *R. solani*; T5—Only *R. solani*; T6—Control (untreated). Data are mean ± Standard Error (*n* = 3) and 95% confidence intervals (*P* < 0.05).

**FIGURE 9:**
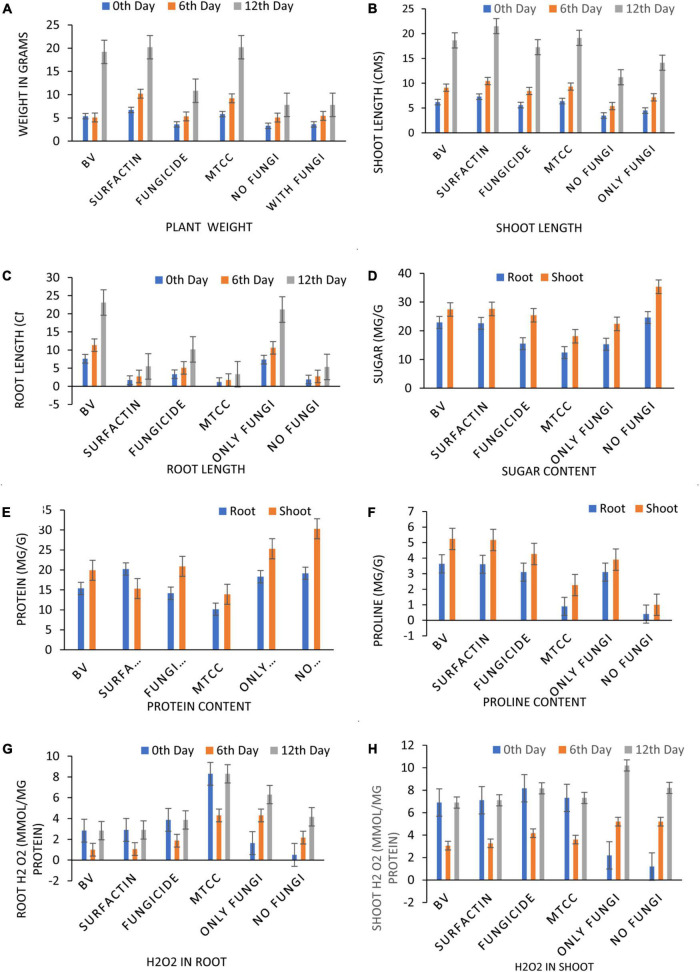
Effects of different treatments on Maize crop **(A)** plant weight, **(B)** shoot length, **(C)** root length, **(D)** total sugar content, **(E)** total protein content, **(F)** total proline, at 7 Days After Pathogen Inoculation (DAPI) and **(G,H)** H_2_O_2_ content in maize roots and shoot at 0, 6th, 12th DAPI under net house condition. Treatments: T1—BV: *B. velezensis* MS20 + *R. solani*; T2—Surfactin + *R. solani*; T3—Fungicide + *R. solani*; T4—*B. subtilis* MTCC2424 + *R. solani*; T5—Only *R. solani*; T6—Control (untreated). Data are mean ± Standard Error (*n* = 3) and 95% confidence intervals (*P* < 0.05).

**FIGURE 10:**
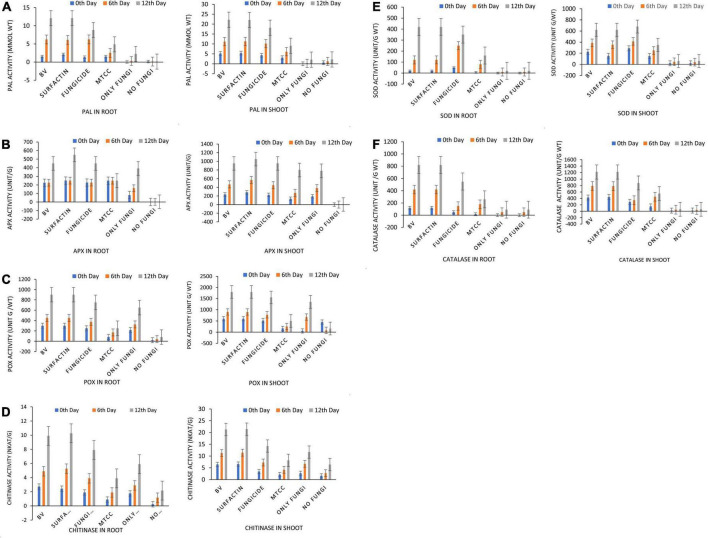
Effects of seed treatments on antioxidant enzyme activity **(A)** phenylalanine ammonia lyase (PAL), **(B)** ascorbate peroxidase (APx), **(C)** peroxidase (POx), **(D)** Chitinase, **(E)** superoxide dismutase (SOD), and **(F)** catalase (CAT) activity in maize root and shoot at 7 DAPI under net house condition. Treatments: T1—BV: *B. velezensis* MS20 + *R. solani*; T2—Surfactin + *R. solani*; T3—Fungicide + *R. solani*; T4—*B. subtilis* MTCC2424 + *R. solani*; T5—Only *R. solani*; T6—Control (untreated). Data are mean ± Standard Error (*n* = 3) and 95% confidence intervals (*P* < 0.05).

Maize seeds coated with MS20 and surfactin (50 μg mL^–1^) after pathogen inoculation revealed that they have significant effect on total chlorophyl content: 10.6 mg g^–1^ fresh weight, carotenoid content 0.46 mg g^–1^ fresh weight, accumulation of protein, proline and sugars (total sugar 22.6, 27.6 mg g^–1^ dry weight, proteins 20.2, 15.3 mg g^–1^ dry weight, and proline 3.6, 5.16 mg g^–1^ dry weight, contents root, and shoot, respectively). Defense enzymes which were detected at an interval of 0, 6th, and 12th days after pathogen inoculation were found to be highest for surfactin i.e., PAL (12.1 μmol trans-cinnamic acid min g^–1^ fresh weight, 22.1 μmol trans-cinnamic acid min g^–1^ fresh weight), APx (550.2 unit g^–1^ fresh weight, 1050.16 unit g^–1^ fresh weight), POx (900.2, 1800.2), H_2_O_2_ 2.9 mmol mg^–1^ protein, 7.1 mmol mg^–1^ protein), SOD (419.9 unit g^–1^ fresh weight., 619.8 unit g^–1^ fresh weight.), CAT (819.9 unit g^–1^ fresh weight., 1219.8 unit g^–1^ fresh weight.), Chitinase (10.2 nKat g^–1^, 21.4 nKat g^–1^), root and shoot, respectively) followed by *B. velezensis* MS20 in comparison to controls (Figures 8–10). From the results it is inferred that MS20 has good plant growth promotion property and its surfactin lipopeptide (50 μg mL^–1^) can be used as a biocontrol agent in maize crop against *R. solani*.

## Discussion

In the present work, a marine bacterium *B. velezensis* MS20 (Ramavath et al., 2019) was used for production of biosurfactant. Marine *Bacillus* are recorded for production of novel bioactive compounds for example lipopeptides, macrolactones, polypeptides, fatty acids, polyketides, and isocoumarins (Mondol et al., 2013). In the present work, a marine bacterium *B. velezensis* MS20 (Ramavath et al., 2019) was used for production of biosurfactant. Among sugars tested, glucose at concentrations lower than 50–60 gL^–1^ is reported to give higher surfactin yield in 48 h (Shaligram and Singhal, 2010; Hmidet et al., 2017). Other than carbon and nitrogen sources, several inorganic nutrients also play a significant role in surfactin lipopeptide production by serving as co-factors for enzymes involved in lipopeptide production (Abdul et al., 2018). In our study, we have deduced that nutrient broth amended with inorganic salt MgSO_4_ and glucose at concentrations of 0.5 and 2% (w/v), respectively, showed the highest surfactin yield of 3,300 mg L^–1^ which is more than the reports on *B. velezensis* KPL2016 which yielded 2,506 mg L^–1^ of surfactin in the presence of 1% w/v glucose (Khem et al., 2018). Our results are in agreement with reports from Hmidet et al. (2017) who reported higher surfactin production at 2% glucose, however, the combination of glucose with MgSO_4_ greatly enhanced yield; a similar type of study was reported for production of surfactin under the influence of MgSO_4_ 2.4 mM concentration by *B. amyloliquefaciens* (Wei et al., 2007; Wibisana et al., 2015). Our results can be supported by a review by Kumar et al. (2021) who discusses the use of molasses and glycerol as “C” source and NH_4_Cl_2_, NH_4_NO_3,_ and NaNO_3_ as an “N” source responsible for high biosurfactant yield.

Upon characterization by TLC, Rf value 0.7 was obtained which is in agreement with recent reports by Parameshwar et al. (2019) in comparison to surfactin standards *srf*AB which is among the four biosynthetic core non-Ribosomal peptide synthetase gene encodes for surfactin lipopeptide (Théatre et al., 2021). In the present study, the PCR amplification of surfactin gene gave strong band at 675 bp which is similar to reports by Swapna et al. (2016). FTIR spectra revealed the presence of functional groups which are characteristic of peptides and aliphatic chains found in surfactin lipopeptide, and LC/ESI-MS peaks showed isoforms normally observed for surfactin, i.e., C12-C16 vibrations in positive and negative polarities which are in accordance with the reports of Table 2 (Sarwar et al., 2018; Janek et al., 2021).

Cyclic lipopeptides from *Bacillus* are reported for their vast therapeutic properties and potential in pharma. Lipopeptide biosurfactants from *B. amyloliquefaciens* and *B. cereus* are known to cause disruption and inhibition of exopolysaccharide gene *Ps1C* expression in *P. aeruginosa* PAO1 cells and other bacteria (Katarzyna et al., 2019). In the present study, surfactin lipopeptide extracted from MS20 was explored for its anti-biofilm activity and it was found to cause aggregation of bacterial pathogens. Bacterial aggregation is usually observed when cell wall disruption occurs due to cleavage of peptidoglycan and prevents colonization. For example, Payne et al. (2013) demonstrated decolonization of *S. aureus* in the presence of tannic acid. Likewise, Rodrigues and Campos-Takaki (2011) and Xiu et al. (2018) have demonstrated the use of lipopeptide in aggregation assay or anti-motility assay for clinical pathogen *Vibrio alginolyticus*178 and *Streptococcus* spp., respectively, in prevention of biofilm formation. In our study, bacterial aggregation assay results inferred visible aggregation for all pathogens by surfactin lipopeptide at MIC 50 μg mL^–1^ concentration.

SEM analysis of MRSA treated with surfactin lipopeptide revealed disruption (50 μg mL^–1^) and aggregation (100 μg mL^–1^). Anti-bacterial and anti-biofilm activity of surfactin against different bacteria has also been documented in a number of studies. For example, at a surfactin concentration of 0.625% w/v, growth inhibition of *Staphylococcus epidermidis* was recorded (Abdelli et al., 2019). Recently, surfactin has been reported to inhibit growth of specific oral pathogens, particularly *S. sanguinis* ATCC105566 at concentrations of > 1.26 × 10^–3^ w/v% (Yamasaki et al., 2020), and removal of biofilms of *Legionella pneumophila* (6.6 × 10^–3^ w/v% of surfactin) (Loiseau et al., 2015). In addition, surfactin is also reported to remove stainless steel and polypropylene surface biofilm of *Listeria monocytogenes, Enterobacter sakazakii*, and *Salmonella enteritidis* (Yamasaki et al., 2020).

*Bacillus* species with a diverse range of bioactive compounds have been identified as sensitizers to control a variety of phytopathogens. The present strain MS20 and its surfactin lipopeptide were found to be effective in limiting the mycelium growth of plant pathogen *R. solani*. Inoculation of actively grown overnight culture of MS20 to PD broth pre-inoculated with *R. solani* resulted in complete inhibition of fungal mycelium in comparison to control. Our results are very much in agreement with recent reports by Teixeira et al. (2021), who demonstrated that *B. velezensis* strain CMRP 4,490 might be used to protect plants as a bio control agent. *In vitro*, *B. velezensis* strain CMRP 4,490 demonstrated strong antagonistic activity against *Sclerotinia sclerotiorum*, *Macrophomina phaseolina*, *Botrytis cinerea*, and *R. solani*. In agriculture, these soil-borne fungus are widespread and difficult to control. As a result, it is essential to develop strategies or solutions to deal with these critical soil-borne fungal infections that cause extensive harm and reduce production of many economically significant crops. Results of this study mirrors those of earlier studies on *B. velezensis* and phytopathogenic fungi (Ge et al., 2016; Xu et al., 2016; Lim et al., 2017). Similarly, Choub et al. (2021) demonstrated that a culture filtrate of *B. velezensis* CE100 displayed appreciable antifungal activity against a phytopathogen (*Colletotrichum gloeosporioides*) which causes anthracnose plant disease.

Generally, the presence of glucose in the fermentation medium is reported to enhance gene expression and can encourage the growth and division of bacteria (Zhou et al., 2015). Likewise, earlier studies have also shown that in the presence of fibers, fever, and high salt in fermentation medium results in selective up-regulation of certain genes to resist exposure to elements in an exigent environment by secretion of some proteins for protection of cells as a defense mechanism (Zhou et al., 2015). A recent q-RTPCR study by Zhou et al. (2018) and Choub et al. (2021) reported lowest fold gene expression (surfactin *sfp* gene) in 1% glucose and highest expression in the presence of a combination of 0.67% glucose and 0.33% cellulose. In the present study we have shown enhanced s*rf*A-A gene expression by q-RTPCR in the presence of 2% glucose and 0.5% MgSO_4_ which upregulated to 9.34 ± 11 -fold in comparison with controls where gene expression was found to be 8-fold less when treated with glucose (1.03 ± 0.1) and MgSO_4_ (1.06 ± 0.1) individually and in untreated controls (1.01 ± 0.1) carbon source; this study can be considered as an early report. Since there are no previously published reports for q-RTPCR surfactin gene expression in the presence of glucose and MgSO_4_, our study should be considered as a preliminary work. However, previously published literature on the effect of glucose on surfactin production states that glucose concentration beyond 50–60 g L^–1^ has a negative effect on surfactin lipopeptide production. Our work will provide a base for future studies in enhanced surfactin yield in the presence and synergism of carbon sources and inorganic mineral salts which cannot be achieved with either of them alone.

Globally, the prevalence of *R. solani*-caused banded leaf and sheath blight disease is on the rise (Li et al., 2019), and it is currently regarded as one of the most destructive diseases of Kharif maize grown in warm and humid regions. At an average temperature of 27–30°C, pathogen *R. solani* becomes more active as relative humidity rises (Hooda et al., 2017; Singh et al., 2020). Seed biopriming triggers ISR effect, enhances germination, helps in uniform establishment of the crop, and fights phytopathogens (Stoll et al., 2021). Given the significance, the goal of this work was to examine if seeds coated with a microbial inoculant activate local and systemic defensive responses in maize against *R. solani*, which causes banded leaf and sheath blight. In the present study, MS20 and its surfactin have showed plant growth promotion as well as biocontrol potential. Plant biocontrol experiment results revealed that maize crop treated with surfactin scored highest in terms of total chlorophyll 10.6 mg g^–1^ fresh weight and carotenoid content 0.46 mg g^–1^ fresh weight in leaves 15 days after pathogen inoculation with *P* < 0.05. Accumulation of biomolecules in root and shoot of maize crop after aforementioned treatments under greenhouse conditions resulted in the highest result for surfactin (total sugar 22.6, 27.6 mg g^–1^ dry weight., proteins 20.2, 15.3 mg g^–1^ dry weight. and proline 3.6, 5.16 mg g^–1^ dry weight contents root and shoot, respectively) as compared to other treatments and control. Likewise, antioxidant enzymes which plants produce as a defense mechanism upon pathogen inoculation to detoxify harmful effect of H_2_O_2_ and reactive oxygen species (ROS), which causes cell death, revealed, increased enzyme production as time progressed in comparison with controls (0 day, 6th day, 12th day) in roots and shoots. Also in root and shoot after treatments, as mentioned in section “Statistical Analysis”, antioxidant enzymes such as PAL (12.1 μmol trans-cinnamic acid min g^–1^ fresh weight, 22.1 μmol trans-cinnamic acid min g^–1^ fresh weight), APx (550.2 unit g^–1^ fresh weight, 1050.16 unit g^–1^ fresh weight), POx (900.2, 1800.2), H_2_O_2_ (2.9 mmol mg^–1^ protein, 7.1 mmol mg^–1^ protein) SOD (419.9 unit g^–1^ fresh weight., 619.8 unit g^–1^ fresh weight.), CAT (819.9 unit g^–1^ fresh weight., 1219.8 unit g^–1^ fresh weight.), and Chitinase (10.2 nKat g^–1^, 21.4 nKat g^–1^) were found to be highly significant i.e., *P* < 0.05 for surfactin as compared to other treatment. Our results on biocontrol activity of *B. velezensis* and surfactin (Kourmentza et al., 2021) against phytopathogen and toward maize crop are comparable and mirrors the reports by Singh et al. (2020) wherein biocontrol efficacy of *P. aeruginosa* MF30, culture supernatant, and culture extract (unidentified) is demonstrated. In the present work, maize seeds treated with surfactin lipopeptide exhibited a significant increase in antioxidant content as well as plant growth in comparison to MS20. Likewise, our results are also in accordance with Liu et al. (2020) wherein *B. velezensis* HC6 and three lipopeptides (iturin, Surfactin, and fengycin) are demonstrated for their potential biocontrol activity in maize crop against phytopathogens *Aspergillus* and *Fusarium* spp. and one pathogenic bacterium, *Listeria monocytogenes*.

## Conclusion

From this work it is concluded that surfactin yield can be enhanced through a combination of a carbon source with a mineral salt MgSO4, and its potential as a biocontrol agent in maize crop for sustainable agriculture is demonstrated. It was also noted to have antibiofilm activity, based on which its application in therapeutics is suggestive.

## Data Availability Statement

The original contributions presented in the study are included in the article/Supplementary Material, further inquiries can be directed to the corresponding author/s.

## Author Contributions

SA and BH: conceptualization. SA, MM, and MK: methodology. SA, BH, and RS: software, validation, investigation, data curation, and writing. BH and RS: formal analysis, visualization, and acquisition. BH: resources, supervision, and project administration. SA, BH, RS, and PP: original draft preparation. BH, RS, MA, AM, and SH: writing—review and editing. PP: reviewing, editing, and revision of the manuscript and acquisition of open access funds. All authors have read and agreed to the published version of the manuscript.

## Conflict of Interest

MK was employed by the company Kalam Biotech Pvt. Ltd. The remaining authors declare that the research was conducted in the absence of any commercial or financial relationships that could be construed as a potential conflict of interest.

## Publisher’s Note

All claims expressed in this article are solely those of the authors and do not necessarily represent those of their affiliated organizations, or those of the publisher, the editors and the reviewers. Any product that may be evaluated in this article, or claim that may be made by its manufacturer, is not guaranteed or endorsed by the publisher.

## References

[B1] Abdelli F. Jardak M. Elloumi J. Stien D. Cherif S. Mnif S. (2019). Antibacterial, anti-adherent and cytotoxic activities of surfactin(s) from a lipolytic strain *Bacillus safensis* F4. *Biodegradation* 30 287–300. 10.1007/s10532-018-09865-4 30600423

[B2] Abdul H. N. Mohd S. M. Lai Y. P. (2018). Culture Medium Development for Microbial-Derived Surfactants Production—An Overview. *Molecules* 23:1049. 10.3390/molecules23051049 29723959 PMC6099601

[B3] Agarwal P. Sharma D. K. (2009). Studies on the production of biosurfactant for the microbial enhanced oil recovery by using bacteria isolated from oil contaminated wet soil. *Pet. Sci. Technol.* 27 1880–1893. 10.1080/10916460802686640

[B4] Akladious S. A. Gomaa E. Z. El-Mahdy O. M. (2019). Efficiency of bacterial biosurfactant for biocontrol of *Rhizoctonia solani* (AG-4) causing root rot in faba bean (Vicia faba) plants. *Eur. J. Plant Pathol.* 153 15–35.

[B5] Armas F. Pacor S. Ferrari E. Guida F. Pertinhez T. A. Romani A. A. (2019). Design, antimicrobial activity and mechanism of action of Arg-rich ultra-short cationic lipopeptides. *PLoS One* 14:212447. 10.1371/journal.pone.0212447 30789942 PMC6383929

[B6] Choub V. Maung C. E. H. Won S.-J. Moon J.-H. Kim K. Y. Han Y. S. (2021). Antifungal Activity of Cyclic Tetrapeptide from *Bacillus velezensis* CE 100 against Plant Pathogen *Colletotrichum gloeosporioides*. *Pathogens* 10:209. 10.3390/pathogens10020209 33672094 PMC7919652

[B7] Das P. Mukherjee S. Sen R. (2008). Antimicrobial potential of a lipopeptide biosurfactant derived from a marine *Bacillus circulans*. *J. Appl. Microbiol.* 104 1675–1684. 10.1111/j.1365-2672.2007.03701.x 18194244

[B8] Díaz De Rienzo M. Banat I. M. Dolman B. Winterburn J. Martin P. (2015). Sophorolipid biosurfactants: possible uses as antibacterial and antibiofilm agent. *New Biotechnol*. 32:009. 10.1016/j.nbt.2015.02.009 25738966

[B9] Ding L. Zhang S. Guo W. Chen X. (2018). Exogenous Indole Regulates Lipopeptide Biosynthesis in Antarctic *Bacillus amyloliquefaciens* Pc3. *J. Microbiol. Biotechnol*. 28 784–795. 10.4014/jmb.1712.12014 29807400

[B10] Ge B. Liu B. Nwet T. T. Zhao W. Shi L. Zhang K. (2016). *Bacillus methylotrophicus* strain NKG-1, isolated from changbai mountain, china, has potential applications as a biofertilizer or biocontrol agent. *PLoS One* 11:e0166079. 10.1371/journal.pone.0166079 27832162 PMC5104391

[B11] Hmidet N. Ayed H. B. Jacques P. Nasri M. (2017). Enhancement of Surfactin and Fengycin Production by *Bacillus mojavensis* A21: application for Diesel Biodegradation. *BioMed. Res. Internat*. 2017 1–8. 10.1155/2017/5893123 29082251 PMC5610860

[B12] Hooda K. S. Khokhar M. K. Parmar H. Gogoi R. Joshi D. Sharma S. S. (2017). Banded Leaf and Sheath Blight of Maize: historical Perspectives, Current Status and Future Directions. *Proc. Natl. Acad. Sci.* 87:1041. 10.1007/s40011-015-0688-5

[B13] Janek T. Gudiña E. J. Połomska X. Biniarz P. Jama D. Rodrigues L. R. (2021). Sustainable Surfactin Production by *Bacillus subtilis* Using Crude Glycerol from Different Wastes. *Molecules* 26:3488. 10.3390/molecules26123488 34201182 PMC8230125

[B14] Jourdan E. Henry G. Duby F. Dommes J. Barthelemy J. P. Thonart P. (2009). Insights into the Defense-Related Events Occurring in Plant Cells Following Perception of Surfactin-Type Lipopeptide from *Bacillus subtilis*. *Am. Phytopathol. Soc.* 22 456–468. 10.1094/MPMI-22-4-0456 19271960

[B15] Katarzyna P. Magdalena M. Grażyna P. Diksha B. Surekha K. S. Przemysław B. (2019). Surfactants of microbial origin as antibiofilm agents. *Int. J. Environ. Res. Public Health* 2019:1664729. 10.1080/09603123.2019.1664729 31509014

[B16] Khem R. M. Tanuja T. Abhishek S. Shamsher S. K. (2018). Lipopeptide antibiotic production by *Bacillus velezensis* KLP2016. *J. Appl. Pharm. Sci.* 8 091–098. 10.7324/JAPS.2018.8313

[B17] Koons B. W. Sobieralski C. A. Comey C. T. Bachtel E. S. Smerick J. B. Presley K. W. (1994). extraction strategies for amplified fragment length polymorphism analysis. *J. Forensic.* 39 1254–1261.

[B18] Kourmentza K. Gromada X. Michael N. Degraeve C. Vanier G. Ravallec R. (2021). Antimicrobial Activity of Lipopeptide Biosurfactants Against Foodborne Pathogen and Food Spoilage Microorganisms and Their Cytotoxicity. *Front. Microbiol.* 11:561060. 10.3389/fmicb.2020.561060 33505362 PMC7829355

[B19] Kumar A. Singh S. K. Kant C. Verma H. Kumar D. Singh P. P. (2021). Microbial Biosurfactant: a New Frontier for Sustainable Agriculture and Pharmaceutical Industries. *Antioxidants* 10:1472. 10.3390/antiox10091472 34573103 PMC8469275

[B20] Li M. S. M. Piccoli D. A. McDowell T. MacDonald J. Renaud J. (2021). Evaluating the biocontrol potential of Canadian strain *Bacillus velezensis* 1B-23 *via* its surfactin production at various pHs and temperatures. *BMC Biotechnol* 21:31. 10.1186/s12896-021-00690-x 33926450 PMC8082884

[B21] Li N. Lin B. Wang H. Li X. Yang F. Ding X. (2019). Natural variation in ZmFBL41 confers banded leaf and sheath blight resistance in maize. *Nat. Genet*. 51 1540–1548. 10.1038/s41588-019-0503-y 31570888

[B22] Lim S. M. Yoon M. Y. Choi G. J. Choi Y. H. Jang K. S. Shin T. S. (2017). Diffusible and volatile antifungal compounds produced by an antagonistic *Bacillus velezensis* G341 against various phytopathogenic fungi. *Plant Pathol. J*. 33 488–498. 10.5423/PPJ.OA.04.2017.0073 29018312 PMC5624491

[B23] Liu Y. Teng K. Wang T. Dong E. Zhang M. Tao Y. (2020). Antimicrobial *Bacillus velezensis* HC6: production of three kinds of lipopeptides and biocontrol potential in maize. *J. Appl. Microbiol*. 128 242–254. 10.1111/jam.14459 31559664

[B24] Loiseau C. Schlusselhuber M. Bigot R. Bertaux J. Berjeaud J. M. Verdon J. (2015). Surfactin from *Bacillus subtilis* displays an unexpected anti-Legionella activity. *Appl. Microbiol. Biotechnol*. 99 5083–5093. 10.1007/s00253-014-6317-z 25573468

[B25] Long X. He N. He Y. Jiang J. Wu T. (2017). Biosurfactant surfactin with pH-regulated emulsification activity for efficient oil separation when used as emulsifier. *Bioresour. Technol.* 241 200–206. 10.1016/j.biortech.2017.05.120 28570884

[B26] Mondol M. A. M. Shin H. J. Islam M. T. (2013). Diversity of Secondary Metabolites from Marine Bacillus Species: chemistry and Biological Activity. *Mar Drugs* 11 2846–2872. 23941823 10.3390/md11082846 PMC3766869

[B27] Parameshwar J. Nageshwar L. Archana K. Bee H. (2019). Enhancement of atrazine biodegradation by marine isolate *Bacillus velezensis* MHNK1 in presence of surfactin lipopeptide. *Ecotoxicol. Env. Safety* 182:109372. 10.1016/j.ecoenv.2019.109372 31255866

[B28] Payne D. E. Martin N. R. Parzych K. R. Rickard A. H. Underwood A. Boles B. R. (2013). Tannic acid inhibits *Staphylococcus aureus* surface colonization in an IsaA-dependent manner. *Infect Immun.* 81 496–504. 10.1128/IAI.00877-12 23208606 PMC3553799

[B29] Ramavath K. Hameeda B. Reddy G. (2019). Enhancement of Plant Growth in Tomato by Inoculation with Plant Growth Promoting *Bacillus* spp. *World J. Agricult. Res.* 7 69–75. 10.12691/wjar-7-2-5

[B30] Rodrigues L. R. M. Campos-Takaki G. M. (2011). Antimicrobial and anti-adhesive potential of a biosurfactant Rufisan produced by *Candida lipolytica* UCP 0988. *Colloids Surf. B. Biointerf*. 84 1–5. 10.1016/j.colsurfb.2010.10.045 21247740

[B31] Sadasivam S. Manickam A. (1996). *Biochemical Methods.* New Delhi: New Age International (P) Ltd, 256.

[B32] Sarwar A. Brader G. Corretto E. Aleti G. Abaidullah M. Sessitsch A. (2018). Qualitative analysis of biosurfactants from *Bacillus* species exhibiting antifungal activity. *PLoS One* 13 1–15. 10.1371/journal.pone.0198107 29864153 PMC5986119

[B33] Shaligram N. S. Singhal R. S. (2010). Surfactin – A Review. *Food Technol. Biotechnol.* 48 119–134.

[B34] Singh S. Singh B. U. Malviya D. Paul S. Sahu P. K. Trivedi M. (2020). Seed Biopriming with Microbial Inoculant Triggers Local and Systemic Defense Responses against *Rhizoctonia solani* Causing Banded Leaf and Sheath Blight in Maize (*Zea mays* L.). *Int. J. Environ. Res. Public Health* 17:1396. 10.3390/ijerph17041396 32098185 PMC7068308

[B35] Staudt C. Horn H. Hempel D. C. Neu T. R. (2004). Volumetric measurements of bacterial cells and extracellular polymeric substance glycoconjugates in biofilms. *Biotechnol. Bioeng*. 88 585–592. 10.1002/bit.20241 15470707

[B36] Stoll A. Salvatierra -Martínez R. González M. Araya M. (2021). The Role of Surfactin Production by *Bacillus velezensis* on Colonization, Biofilm Formation on Tomato Root and Leaf Surfaces and Subsequent Protection (ISR) against Botrytis cinerea. *Microorganisms* 9:2251. 10.3390/microorganisms9112251 PMC8626045 34835375

[B37] Swapna T. H. Papathoti N. K. Khan M. Y. Reddy G. Bee H. (2016). Bioreduction of Cr (VI) by biosurfactant producing marine bacterium *Bacillus subtilis* SHB 13. *J. Sci. Ind. Res.* 75 432–438.

[B38] Teixeira G. M. Mosela M. Nicoletto M. L. A. Ribeiro R. A. Hungria M. Youssef K. (2021). Genomic Insights Into the Antifungal Activity and Plant Growth-Promoting Ability in *Bacillus velezensis* CMRP 4490. *Front. Microbiol*. 11:618415. 10.3389/fmicb.2020.6184 PMC7844144 33519779

[B39] Théatre A. Cano-Prieto C. Bartolini M. Laurin Y. Deleu M. Niehren J. (2021). The Surfactin-Like Lipopeptides From *Bacillus* spp.: Natural Biodiversity and Synthetic Biology for a Broader Application Range. *Front. Bioeng. Biotechnol.* 9:623701. 10.3389/fbioe.2021.623701 33738277 PMC7960918

[B40] Thimmaiah S. R. (2012). *Standard Methods of Biochemical Analysis.* New Delhi: Kalyani Publishers, 421–426.

[B41] Vatsa P. Sanchez L. Clement C. Baillieul F. Dorey S. (2010). Rhamnolipid biosurfactants as new players in animal and plant defense against microbes. *Int. J. Mol. Sci.* 11 5095–5108. 10.3390/ijms11125095 21614194 PMC3100842

[B42] Wei Y. H. Lai C. C. Chang J. S. (2007). Using Taguchi experimental design methods to optimize trace element composition for enhanced surfactin production by Bacillus subtilis ATCC 21332. *Proc. Biochem.* 42 40–45. 10.1016/j.procbio.2006.07.025

[B43] Wibisana A. Sumaryono W. Sudiro T. Sudarmono P. (2015). Optimization of Surfactin Production by *Bacillus amyloliquefaciens* MD4-12 Using Response Surface Methodology. *Microbiol. Indon.* 9 120–128. 10.5454/mi.9.3.4 25265523

[B44] Xiu P. Liu R. Zhang D. Sun C. (2018). Bacterial Aggregation Assay in the Presence of Cyclic Lipopeptides. *Bio-protocol*. 8:e2686. 10.21769/BioProtoc.2686 34179236 PMC8203917

[B45] Xu T. Zhu T. Li S. (2016). β-1,3-1,4-glucanase gene from *Bacillus velezensis* ZJ20 exerts antifungal effect on plant pathogenic fungi. *World J. Microbiol. Biotechnol*. 32:26. 10.1007/s11274-015-1985-0 26745986

[B46] Yamasaki R. Kawano A. Yoshioka Y. Ariyoshi W. (2020). Rhamnolipids and surfactin inhibit the growth or formation of oral bacterial biofilm. *BMC Microbiol.* 20:358. 10.1186/s12866-020-02034-9 33228524 PMC7684882

[B47] Zhou Z. Liu F. Zhang X. Zhou X. Zhong Z. Su H. (2018). Cellulose-dependent expression and antibacterial characteristics of surfactin from *Bacillus subtilis* HH2 isolated from the giant panda. *PLoS One* 13:e0191991. 10.1371/journal.pone.0191991 29385201 PMC5791997

[B48] Zhou Z. Zhou X. Li J. Zhong Z. Li W. Liu X. (2015). Transcriptional regulation and adaptation to a high-fiber environment in *Bacillus subtilis* HH2 isolated from feces of the Giant Panda. *PLoS One* 10:e0116935. 10.1371/journal.pone.0116935 25658435 PMC4319723

