# Supplementary material for: Induction of Systemic Resistance in Maize and Antibiofilm Activity of Surfactin From Bacillus velezensis MS20
Source: Front Microbiol. 2022 May 9;13:879739. doi: 10.3389/fmicb.2022.879739 (PMC9126211; doi:10.3389/fmicb.2022.879739)
Supplement: Supplementary file 1 [file Data_Sheet_1.docx]

| **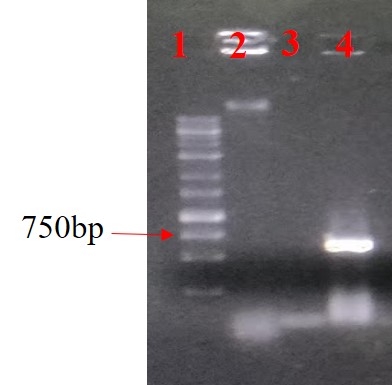**  **S1:** PCR amplification of *srfAB*gene in *B. velezensis* MS20, Lane.1: 1KB Ladder, Lane.2: Genomic DNA, Lane.3: Control, Lane. 4: Test *srfAB* gene. Band at approx. 675bp [20] |
| --- |
| **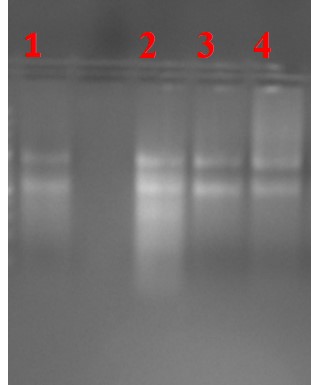** |
| **S2:** Quantification of RNA of *B. velezensis* MS20:  Lane. 1: Treatment 1 (NB untreated),  Lane. 2: Treatment 2 (NB+0.5 % MgSO4).  Lane. 3: Treatment 2 (NB + 2 % Glucose),  Lane. 4: Treatment 2 (NB +2 % Glucose + 0.5% MgSO4). |
